# Language and cognitive function in children: a narrative review of neural, behavioral, and developmental evidence

**DOI:** 10.3389/fpsyg.2025.1666719

**Published:** 2025-08-29

**Authors:** Ling Pu, Sergey Kiselev, Ningkun Xiao

**Affiliations:** ^1^Laboratory for Brain and Neurocognitive Development, Department of Psychology, Institution of Humanities, Ural Federal University, Yekaterinburg, Russia; ^2^Department of Immunochemistry, Institution of Chemical Engineering, Ural Federal University, Yekaterinburg, Russia

**Keywords:** language, cognition, cross-culture, cognitive development, language cognition

## Abstract

Language is not merely a conduit for thought-it plays an active, constitutive role in shaping cognitive development. This narrative review synthesizes interdisciplinary findings across bilingualism, theory of mind, developmental disorders (DLD and ASD), and cross-cultural studies to propose a dynamic, context-sensitive model of the language-cognition relationship. We argue that language functions not only as a cognitive tool but as a cognitive architect, influencing the structure and function of neural networks supporting executive function and social cognition. Evidence from behavioral and neuroimaging studies reveals bidirectional and developmentally contingent interactions between language and cognition, moderated by linguistic structure, developmental timing, and sociocultural context. By examining both typical and atypical populations, we challenge modular and unidirectional models, advocating instead for integrative frameworks that capture the diversity and plasticity of human cognition. We conclude with a roadmap for future research, emphasizing longitudinal, cross-linguistic, and translational approaches. This work calls for a rethinking of language's role-not as a passive reflection of mind, but as its formative force.

## 1 Introduction

Language is one of the most distinctive achievements of human cognition. Beyond serving as a medium for social communication, it functions as a central tool in shaping thought, organizing experience, and regulating behavior (Lupyan, 2016). Traditional views in cognitive science have often framed language as a modular system subject to top-down regulation by domain-general Executive Function (EF) (Shokrkon and Nicoladis, 2022). However, a growing body of neuroscientific, behavioral, and developmental research has challenged this unidirectional control model. Instead, evidence increasingly supports a bidirectional and deeply interwoven relationship between language and cognitive functions (Perszyk and Waxman, 2018; Goldin-meadow et al., 2014).

Early linguistic experience—encompassing the quantity, quality, and diversity of input (e.g., monolingual, bilingual, and multilingual environments)—exerts profound and lasting influence on the trajectory of cognitive development, including executive function, working memory, and social cognition (Shokrkon and Nicoladis, 2022; Garfield et al., 2001). This implies that language is not merely a communicative tool but a core architect of cognitive development. Understanding how language evolves from a passive medium into an active constructor of cognition is essential for uncovering the developmental foundations of the human mind.

In this review, we aim to synthesize recent progress on the interaction between language and cognition during childhood, moving beyond modular frameworks toward a dynamic, integrative perspective. Specifically, we seek to:

1. Examine the co-developmental relationship between language and executive function;
2. Use the “bilingual advantage” debate to highlight methodological and moderating factors in the study of language-cognition interactions;
3. Investigate the scaffolding role of language—particularly syntax—in the development of higher-order social cognition such as theory of mind;
4. Analyze patterns of co-occurring deficits in language and cognition in populations with developmental language disorder (DLD) and autism spectrum disorder (ASD) to elucidate their deep interdependence;
5. Emphasize the importance of cross-linguistic and cross-cultural research in testing the universality of language–cognition theories.

By reviewing these key domains, we aim to provide a scientific foundation for improving educational policy (e.g., bilingual education), advancing clinical interventions for language and cognitive disorders, and deepening theoretical insights into the architecture of human cognition.

## 2 The Core engine: the co-development of language and executive function

The relationship between language and EF represents a foundational topic in cognitive science. EF refers to a set of higher-order regulatory processes that support goal-directed behavior, including working memory, inhibitory control, and cognitive flexibility (Friedman and Robbins, 2022; Zelazo, 2015). Traditional models have tended to conceptualize EF as a central controller that exerts top-down regulation on domain-specific systems, including language (Fedorenko, 2014). However, emerging longitudinal and neuroimaging evidence suggests a more reciprocal, co-developer relationship in which language and EF evolve interactively throughout development (Shokrkon and Nicoladis, 2022; Gooch et al., 2016).

These dynamic systems view call for a reassessment of how these domains interact across developmental time and offer a more integrated framework for understanding both typical and atypical cognitive development.

### 2.1 Beyond unidirectionality: toward a bidirectional developmental framework

A growing number of longitudinal studies support a bidirectional influence between language and EF. Some findings indicate that early language abilities can predict later improvements in EF. For instance, Kuhn et al. found that the rate of growth in expressive language abilities (vocabulary size and syntactic complexity) between 15 and 36 months significantly predicted gains in EF from 36 to 60 months, as well as EF levels at age five (Kuhn et al., 2016). These results align with Vygotskian theory, which posits that early language serves as a symbolic tool for the development of self-regulation and executive control (Vallotton and Ayoub, 2011).

Conversely, there is also evidence that early advances in EF facilitate subsequent language growth (Ekerim and Selcuk, 2018). Improvements in inhibitory control during toddlerhood, (e.g.,), have been linked to greater vocabulary gains, and performance on interference suppression tasks (e.g., the Stroop task) at age five has been found to predict syntactic proficiency (Bub et al., 2006; Ibbotson and Kearvell-white, 2015). These findings support a developmental cascade model, in which the two systems grow in parallel and mutually reinforce one another over time.

Within this cascade framework, progress in one domain creates scaffolding for advancement in the other. For example, rapid vocabulary acquisition may provide children with representational tools for performing complex rule-based tasks, while enhanced inhibitory control may free cognitive resources needed for mastering hierarchical syntactic structures. Such intertwined developmental trajectories imply that interventions should be dynamically aligned with children's developmental stages. When language and EF are reciprocally reinforcing, isolated intervention in one domain may be insufficient; instead, adaptive strategies that jointly target both systems may yield more robust outcomes.

### 2.2 Shared neural foundations: overlapping networks and co-activation

The bidirectional relationship between language and EF, evident at the behavioral level, is also mirrored in the brain (Shokrkon and Nicoladis, 2022; Lehtonen et al., 2023). Rather than operating as distinct, modular systems, language and EF rely on partially overlapping and tightly interconnected neural networks. This perspective departs from classical modular models of language, such as the Broca–Wernicke framework (Nasios et al., 2019), and aligns with modern network-based views of brain organization (Chang et al., 2015).

One particularly relevant system is the multiple-demand (MD) network, which is recruited across a wide range of cognitively demanding tasks (Cai et al., 2024). Neuroimaging studies have shown that early balanced bilinguals exhibit stronger activation of the MD network during executive processing tasks compared to monolinguals (Malik-moraleda et al., 2021; Grundy et al., 2017). For example, a large-scale fMRI study revealed that bilinguals demonstrated significantly greater MD system activation during spatial working memory tasks, directly linking bilingual experience to the neural architecture of cognitive control (Malik-moraleda et al., 2021). These findings provide neurobiological evidence for how language experience can shape executive systems.

Moreover, during complex linguistic tasks—such as syntactic ambiguity resolution—or language tasks that require cognitive control, traditional language-related regions (e.g., the left inferior frontal gyrus) and prefrontal executive regions are co-activated (Ye and Zhou, 2009; Coderre et al., 2016). This overlapping activation pattern suggests that language processing is not supported by a fixed set of cortical areas but rather by dynamically assembled functional alliances. For relatively simple and automatic language tasks, activation is confined largely to perisylvian regions; however, for more complex or control-demanding language operations—such as code-switching or interpreting puns—the brain recruits broader resources from the MD system.

Lesion-symptom mapping studies in individuals with aphasia also support this non-modular account. Damage to Broca's area is frequently associated not only with impaired language production but also with deficits in nonverbal cognitive control (Fedorenko et al., 2012). This indicates that Broca's area contains subregions with distinct functions: some are highly specialized for language, while others participate in domain-general control processes (Bulut, 2023). Studies have visually demonstrated this distinction, showing that language-selective and domain-general regions are juxtaposed within Broca's area (Fedorenko et al., 2012; Fedorenko and Blank, 2020). The former are selectively activated during sentence comprehension, whereas the latter are engaged across various cognitive tasks (Fedorenko et al., 2012).

Thus, from a neural standpoint, language processing and executive control are not cleanly separable; in many contexts, the controlled use of language is itself an act of executive function (Ye and Zhou, 2009). This also explains why lesions in traditionally defined “language areas” sometimes lead to EF impairments. For instance, patients with focal damage to Broca's area often show not only expressive aphasia but also marked difficulties in task switching, inhibitory control, and verbal working memory—key components of executive functioning (Irani et al., 2025; Fedorenko et al., 2012). These findings suggest that parts of Broca's area contribute to domain-general control processes that extend beyond language, reinforcing the view that language and executive control rely on shared neural substrates rather than distinct modular systems. From an evolutionary perspective, language-related brain regions such as Broca's and Wernicke's areas exhibit distinct anatomical specializations in humans, including broader and more widely spaced cortical minicolumns (Rilling, 2008; Buxhoeveden et al., 2001), as well as increased horizontal connectivity and cytoarchitectonic complexity (Zimmermann et al., 2024; Galakhova et al., 2022). These structural features suggest that the evolution of language was accompanied by a reorganization of existing domain-general neural systems, supporting the view that language functions emerge from, and are integrated with, broader cognitive control architecture.

### 2.3 Working memory: a central interface between language and cognition

Working Memory (WM), particularly verbal working memory, represents a critical interface through which language interacts with other cognitive processes (Baddeley, 2003). It is important to recognize that WM capacity and efficiency are not merely properties of fixed “cognitive hardware” but are significantly shaped by an individual's linguistic knowledge (Miyake and Friedman, 2013).

Empirical studies show that verbal WM performance leverages long-term representations of phonological, lexical, and semantic patterns (Schwering and Macdonald, 2020). Participants consistently recall real words more accurately than pseudo words and perform better with pseudo words that resemble real words in sound structure than with those that do not (Messer et al., 2010). In other words, working memory is not a passive buffer; it actively recruits stored language knowledge to enhance performance (Messer et al., 2010).

This phenomenon has significant implications for bilingual assessments. Bilinguals often have smaller vocabularies in each language compared to monolinguals, which can disadvantage them in verbal memory tasks administered in a single language (Bialystok, 2009; Kaushanskaya et al., 2011). However, this does not indicate weaker cognitive capacity—rather, it reflects unfair demands on their lexical resources (Bialystok, 2009). Indeed, studies have found that bilinguals may underperform monolinguals in rote repetition tasks but perform equivalently in tasks that require cognitive control (Bialystok, 2009). This suggests that lower performance on traditional verbal tests may reflect vocabulary differences rather than true disparities in memory ability.

Research on developmental language disorder (DLD) further highlights the role of verbal WM. One of the hallmark cognitive impairments in DLD is a persistent and severe deficit in verbal working memory (Niu et al., 2024). Children with DLD typically underperform on tasks involving digit span, word span, and sentence repetition (Larson and Ellis weismer, 2022). This WM deficit is regarded as a defining cognitive feature of DLD. In contrast, findings regarding nonverbal EF in DLD are more heterogeneous: while some studies report difficulties in attention control and task switching, others do not, and considerable variability exists within this population (Montgomery, 2002; Conti-ramsden et al., 2015). This imbalance has led to a consistent pattern: verbal WM is reliably impaired, whereas nonverbal EF is variable and less consistently affected.

These findings challenge single-deficit models of DLD. If EF deficits were the root cause, nearly all children with DLD should exhibit such impairments, yet many demonstrate typical performance on nonverbal WM and executive tasks. A more plausible account is that language and EF are mutually influential developmental systems, and their dysregulation jointly contributes to the cognitive phenotype observed in DLD (Niu et al., 2024).

Taken together, these lines of evidence support the notion that language and EF are embedded in a recursive feedback loop from early development. Language experience shapes the architecture of cognitive control networks. Conversely, executive functions—such as attentional control, cognitive flexibility, and inhibitory regulation—can both support and limit language development, depending on their efficiency and maturation (Diamond, 2013; Jurado and Rosselli, 2007). For example, sustained attention and working memory capacity facilitate vocabulary acquisition and sentence parsing, while deficits in inhibitory control may impede the suppression of irrelevant linguistic information or hinder discourse planning (Novick et al., 2010; Korko and Williams, 2017). In this way, executive capacities provide the scaffolding for language to develop efficiently—but when underdeveloped, they may also impose constraints. Rather than running on parallel tracks, language and executive function form an interwoven, co-regulated system. As we emphasize throughout this review, language is not merely involved in cognition—it helps construct it.

## 3 Deconstructing the “bilingual executive function advantage” debate

The bilingual advantage hypothesis proposes that managing two languages on a daily basis strengthens domain-general executive function (EF) through continuous practice in control, inhibition, and selection (Paap et al., 2025). Over the past two decades, this idea has evolved into one of the most intensely debated and methodologically scrutinized topics in cognitive science (Malik-moraleda et al., 2021).

Several empirical studies have reported enhanced performance among bilinguals in tasks involving attentional control and conflict resolution, such as the Simon task and the Attention Network Test (Bialystok et al., 2004; Costa et al., 2008), and several meta-analyses have concluded moderate advantages for bilinguals under certain conditions (Adesope et al., 2010; Gunnerud et al., 2020). Proponents argue that bilingual language use enhances domain-general EF by repeatedly engaging mechanisms for monitoring, switching, and inhibition (Lehtonen et al., 2023; Wu et al., 2021; Bialystok, 2011).

However, a growing body of evidence has challenged the robustness and generalizability of these effects. Critics emphasize inconsistent replication, task impurity, and potential confounds such as socioeconomic status, immigration status, and publication bias (Paap and Greenberg, 2013; Lehtonen et al., 2018; De bruin et al., 2015). Other meta-analyses have found negligible or null effects across diverse cognitive tasks (Donnelly et al., 2019).

These conflicting findings have shifted the focus of the field from a binary question of presence or absence of a bilingual advantage to a more nuanced consideration of boundary conditions, measurement reliability, and individual difference factors (Grundy and Timmer, 2017; Valian, 2015). As such, the debate increasingly reflects a deeper methodological and theoretical divide within developmental cognitive neuroscience.

### 3.1 The rise and re-evaluation of the bilingual advantage hypothesis

The bilingual advantage hypothesis gained early momentum with pioneering studies demonstrating that managing two languages may bolster domain-general EF—such as inhibitory control and task switching—through ongoing cognitive practice (Bialystok et al., 2004; Costa et al., 2008). Supportive findings extended across development: bilingual children outperformed monolingual peers in conflict-monitoring tasks (Costa et al., 2008; Novitskiy et al., 2019), while lifelong bilingualism appeared to delay the onset of age-related cognitive decline in older adults (Bialystok, 2011; Bialystok et al., 2016).

As the literature grew, meta-analytic efforts began to probe the robustness of the advantage. Some syntheses reported small to moderate bilingual benefits under certain conditions—for instance, Adesope et al. (Adesope et al., 2010) observed modest overall EF gains, and De bruin et al. (2021) found a global EF advantage. However, accumulating evidence also challenged the generalizability of the advantage. Critics identified methodological shortcomings, including publication bias and confounding variables like socioeconomic status and immigration history (De bruin et al., 2015; Paap and Greenberg, 2013; Lehtonen et al., 2018). Other meta-analyses reported negligible or null effects across age groups (Donnelly et al., 2019; Gunnerud et al., 2020).

Taken together, the field has evolved from a simple “yes/no” framing of the bilingual advantage to recognizing a context-dependent phenomenon. Now, advantage appears contingent on factors such as participant age, task type, proficiency level, and study quality (Grundy and Timmer, 2017; Ware et al., 2020). Consequently, the debate has shifted toward addressing methodological rigor and boundary conditions, rather than proving or disproving a universal effect. And the bilingual advantage debate has evolved into a nuanced scientific inquiry where both supportive and contradictory findings have advanced our understanding of the interplay between bilingual experience and executive function. It emphasizes that bilingualism may support executive control under specific circumstances—particularly in tasks with high conflict demands or in populations with greater cognitive plasticity (e.g., children, older adults)—but it should not be assumed as a general cognitive enhancer. Future research must refine measurement paradigms, control for confounds, and delineate when and for whom bilingual experience yields EF benefits.

### 3.2 Identifying moderators: the “when, who, and what” of bilingual advantage

A growing consensus holds that any putative bilingual advantage is contingent rather than universal, and it varies along the when—who—what dimensions. When bilingual experience relates to EF appears to depend on developmental stage and everyday interactional contexts. Reported group differences are more frequent in older adults than in young adults—consistent with cognitive-reserve or adaptation accounts—whereas findings in children and young adults are typically small or null unless tasks impose substantial conflict-monitoring or switching demands (Bialystok et al., 2012, 2024; Ware et al., 2020). Moreover, interactional context matters: more integrated dual-language use and higher switching frequency are associated with reduced switch costs and modest benefits on control-demanding paradigms, in line with the Adaptive Control Hypothesis and usage-based perspectives (Green and Abutalebi, 2013; Prior and Gollan, 2011; Hartanto and Yang, 2016).

Who appears to benefit reflects individual profiles, but not in a threshold-like way. Higher proficiency across both languages and more frequent dual-language use have been associated with slightly larger effects in some studies; however, converging work suggests that what better predicts outcomes is the intensity and diversity of control demands—indexed by integrated usage patterns, habitual language switching, and language-use entropy—rather than proficiency alone (Iniesta et al., 2025; Pliatsikas, 2020; Van den berg et al., 2022). Background factors such as socioeconomic status and immigration history can attenuate, inflate, or even reverse apparent group differences (Morton and Harper, 2007; Calvo and Bialystok, 2014). Importantly, within a bidirectional framework, monolingual language experience (e.g., lexical growth, syntactic proficiency) also correlates with EF components (e.g., interference control, updating, controlled parsing), supporting a general experience-dependent adaptation account whereby language use—mono- or bilingual—shapes attention/control systems through practice (Novick et al., 2010; De abreu et al., 2011).

What is observed depends on EF components, task features, and measurement quality. Moderator analyses and bias-corrected syntheses indicate that small, relatively stable differences sometimes appear for interference control (e.g., Flanker/Stroop), whereas purported effects on response inhibition (e.g., Go/No-Go) and working memory/updating are less reliable and often vanish after correcting for publication bias (Paap and Greenberg, 2013; Donnelly et al., 2019; Paap et al., 2020; Ware et al., 2020; Lehtonen et al., 2018). Some overviews further suggest that group differences may reflect processing-speed differences rather than core EF *per se*, and concerns about task impurity, psychometric reliability, and analytic flexibility constrain generalization (Lehtonen et al., 2018; Hedge et al., 2018). Taken together, where bilingual—monolingual differences are detected, they appear more likely under specific developmental and usage contexts, for particular EF components, and with appropriately sensitive and reliable tasks—patterns that align more closely with experience-dependent adaptation to specific control demands than with a broad, domain-general enhancement.

In sum, current evidence suggests that potential bilingual advantages are contingent on a complex interplay of factors, including developmental stage, task type, executive function domain, proficiency level, and patterns of language use. Such advantages seem to emerge most consistently when multiple facilitating conditions converge, rather than as a universal by-product of bilingualism.

Yet, even when carefully considering these moderating variables, the observed variability in outcomes remains striking. This raises a critical question: to what extent do the mixed results in the literature reflect true heterogeneity in the underlying cognitive effects, and to what extent might they be shaped by the way research is disseminated and reported? Addressing this question requires examining structural influences on the evidence base—most notably, the role of publication bias.

### 3.3 The shadow of publication bias

An often under examined factor in the bilingual advantage debate is publication bias—the tendency for studies reporting significant effects to be more likely published than those reporting null results. Several studies have documented this skew in the bilingual advantage literature (Lehtonen et al., 2018; De bruin et al., 2015; Donnelly et al., 2019). For example, De bruin et al. (2015) surveyed conference abstracts and found that studies with null results were substantially less likely to appear in peer-reviewed journals.

While this pattern suggests that the published record may represent only the “visible tip” of a larger body of mixed or null findings, it is important to acknowledge recent structural shifts in dissemination practices that actively counteract this bias. The growth of preprint servers and open data repositories has enabled the rapid sharing of null and replication results, increasing transparency and reducing the dependence on traditional journal gatekeeping (Tracz and Lawrence, 2016; Smart, 2022).

Consequently, any assessment of the bilingual advantage hypothesis must consider publication bias not merely as a methodological artifact but as a shaping force in the scientific narrative—one that is itself evolving. The critical question may not be whether bilingualism confers cognitive benefits in principle, but how our research ecosystem, including incentives, publication practices, and open-science initiatives, determines which outcomes are amplified, which are muted, and how the balance between them changes over time.

### 3.4 From “advantage” to “adaptation”: a neurocognitive perspective

In light of the variability and ambiguity in behavioral results, many scholars have moved beyond the binary “advantage or no advantage” question to adopt a more mechanistic view: bilingual experience as a driver of neurocognitive adaptation. This perspective aligns with the well-established notion of language as a potent neuroplasticity-inducing factor—an idea long recognized in cognitive neuroscience (Baum and Titone, 2014; Bialystok et al., 2014; Del maschio et al., 2018; Korenar et al., 2023). “Cognitive architect” is used as a metaphor to describe how bilingualism, through sustained engagement with two linguistic systems, continuously remodels neural circuitry to meet the unique demands of bilingual communication.

Neuroimaging studies show that bilinguals often display structural and functional differences in regions of the MD network, with greater recruitment during executive EF tasks compared to monolinguals (Pliatsikas and Luk, 2016). Electrophysiological evidence similarly reveals distinct event-related potential (ERP) patterns during conflict monitoring and resolution (Botezatu et al., 2021), pointing to changes in neural efficiency or processing strategies. Crucially, such adaptations are robust and replicable even when behavioral differences are small or absent, suggesting that neural reconfiguration may precede, or occur independently of, overt performance changes.

This neurocognitive adaptation framework helps to resolve a long-standing paradox: the frequent observation of neural differences in the absence of strong behavioral effects. The explanation lies in the reorganization of cognitive control systems to handle challenges such as language switching, interference suppression, and cross-linguistic monitoring. These adaptations may optimize brain function for bilingual contexts without necessarily producing generalized EF advantages on laboratory tasks (Bialystok, 2017).

Thus, the central question is not whether bilinguals are universally “better,” but how bilingual experience interacts with neuroplastic mechanisms to shape the architecture of cognitive control. In this view, bilingualism represents a powerful experiential context that, much like other forms of intensive cognitive engagement, continuously shapes neural and cognitive landscapes over the lifespan.

## 4 How language shapes higher-order cognition

One of the hallmarks of human cognition is the ability to reason about one's own and others “mental states—a capacity known as Theory of Mind (ToM) (Siegal and Varley, 2002). The ability to understand others” beliefs, desires, and intentions represents a key milestone in the development of social cognition (Frith and Frith, 2012). Yet, how children acquire ToM has long been debated. While some accounts emphasize innate modules or conceptual leaps, others increasingly highlight the constructive role of language in scaffolding the development of social understanding (De villiers and De villiers, 2025).

### 4.1 From words to worlds: the bidirectional link between language and social cognition

The development of ToM does not occur in isolation; rather, it is intricately intertwined with language acquisition (Garfield et al., 2001). From infancy onward, this relationship is bidirectional. On the one hand, early ToM skills—such as understanding others' intentions—can support language learning. For instance, infants who attend to intentional actions (e.g., purposeful gestures or pointing) are better at mapping words to referents (Tomasello et al., 2005). Research has shown that infants can differentiate between intentional and accidental actions and preferentially imitate goal-directed behaviors (Elsner, 2007). This indicates that early mentalizing abilities are already guiding vocabulary acquisition.

On the other hand, as children mature, language begins to serve as a powerful tool for navigating and reasoning about the social world. Children can already use linguistic cues to infer a speaker's knowledge state or epistemic certainty (Hübscher et al., 2019). For example, toddlers interpret “I know X” as more confident than “I think X,” suggesting that children use subtle semantic distinctions to infer others' beliefs and attitudes.

Overall, language and social cognition co-develop through a dynamic, reciprocal process. Language provides a medium for expressing and rehearsing mental state reasoning, while social cognition directs attention and motivation during language learning. The development of perspective-taking and intention understanding is thus closely tied to the growth of linguistic competence.

### 4.2 The syntax key: unlocking false-belief reasoning

Why do children only reliably pass standard false-belief tasks around age four (Hughes et al., 2000)? One influential account emphasizes the acquisition of a specific syntactic structure—sentential complements (De villiers, 2005). These embedded clauses allow the expression of beliefs that may diverge from reality, e.g., “Mary thinks the ball is in the box.” Mastering such constructions has been linked to the ability to represent others' mistaken beliefs, with longitudinal and training studies showing that early competence in complement clauses predicts earlier success in false-belief reasoning (De villiers and De villiers, 2025; Boeg Thomsen et al., 2021; Mo et al., 2014). This association remains robust even after controlling for vocabulary size, memory span, and general language ability (Lind and Bowler, 2009).

The prevailing interpretation is that complement syntax provides the representational format needed to mentally simulate propositions that contradict reality, thereby scaffolding mature Theory of Mind (ToM). However, alternative interpretations remain viable. As some scholars argue, the use of such syntax may function less as a causal enabler and more as a behavioral marker of underlying cognitive readiness for ToM (Baillargeon et al., 2010). Language does not necessarily create the capacity to distinguish between personal knowledge and others' misbeliefs but serves as an observable artifact—a socially available tool that makes such distinctions communicable and testable in experimental contexts.

Recognizing both perspectives helps clarify the current debate. On one hand, complement syntax may facilitate the mental representation of false beliefs; on the other, its emergence in children's speech could simply coincide with, and reveal, a pre-existing maturation of ToM-related cognitive architecture. Distinguishing between these possibilities remains an open empirical challenge, which points to the importance of integrative approaches that combine linguistic, cognitive, and neurodevelopmental evidence.

### 4.3 New methods for tracking neurodevelopmental trajectories

Recent advances in child-friendly neuroimaging methods—such as functional near-infrared spectroscopy (fNIRS) and high-density EEG—have opened a new window into the neural underpinnings of language and higher-order cognition in early development.

fNIRS has proven to be a sensitive and non-invasive tool for measuring cortical activation in infants and young children during exposure to various sensory and social stimuli (Quaresima and Ferrari, 2019). For example, converging evidence from developmental neuroscience shows that even in the first year of life, infants' temporal and frontal cortices respond selectively to socially relevant cues such as eye gaze, facial expressions, and infant-directed speech (Grossmann and Johnson, 2007; Grossmann, 2015). These neural responses are thought to support early social attention and may form a foundation upon which more complex communicative skills later emerge, although the link to communicative intent *per se* requires careful, task-specific testing.

Moreover, a longitudinal fNIRS study of 4-year-olds found that resting-state functional connectivity between the prefrontal cortex and other regions predicted individual differences in executive function months later (Kerr-german et al., 2022). These findings indicate that the maturity of early brain networks plays a role in shaping later cognitive development.

Moving forward, integrating multiple neuroimaging modalities holds promise for a more comprehensive understanding of the language-cognition interface. Combining the high temporal resolution of EEG with the moderate spatial resolution of fNIRS, for example, enables researchers to simultaneously track the timing and localization of brain activity during cognitive tasks. This multimodal approach can help identify the precise moments and neural circuits involved when children deploy language to reason about others' minds.

Such methods offer exciting potential for testing causal hypotheses—such as whether learning sentential complements leads to measurable changes in neural activation during ToM tasks. By capturing the dynamic interplay between linguistic input and cognitive development, these tools will allow researchers to map how the “neural architecture of social cognition” is constructed in real time.

## 5 Insights from the margins: atypical development as a window into cognitive architecture

One powerful method for investigating the architecture of the cognitive system is to observe how it manifests under atypical developmental conditions. Developmental disorders—such as DLD and Autism Spectrum Disorder (ASD)—present not only clinical challenges but also serve as valuable “natural experiments.” These populations often display non-canonical patterns of interaction or dissociation between language and cognition, offering rare opportunities to reveal connections that are otherwise tightly intertwined and difficult to tease apart in typical development.

DLD is characterized by persistent difficulties in language acquisition (Montgomery, 2002); ASD is marked by deficits in social communication and restricted, repetitive behaviors (Xiao et al., 2024). Yet, both are cognitively complex conditions in which impairments in language and EF frequently co-occur. By comparing these distinct atypical profiles, we can test the limits of modular accounts and gain deeper insight into the developmental interdependence of language and cognition.

### 5.1 DLD: a language deficit, a cognitive deficit, or both?

Formerly known as Specific Language Impairment (SLI), the condition now referred to as Developmental Language Disorder (DLD) has undergone substantial terminological refinement in recent years. A major step in this process—a consensus study led by Bishop et al. (Bishop et al., 2017a,b) and involving an international panel of experts—which recommended adopting the term DLD to improve diagnostic clarity and cross-disciplinary communication. The change was motivated by persistent challenges in defining the disorder solely in terms of exclusionary criteria (e.g., absence of hearing loss, intellectual disability) and by the recognition that language impairments often co-occur with other cognitive difficulties (Bishop et al., 2017b,a).

Historically, DLD was conceptualized as an isolated deficit in language (Montgomery, 2002). However, accumulating evidence reveals that DLD is rarely a “pure” language impairment—it often coexists with broader cognitive difficulties, particularly in verbal memory and processing (Montgomery, 2002; Clegg et al., 2005). Children with DLD consistently perform poorly on verbal working memory (WM) tasks across languages and ages (Jackson et al., 2021). These deficits are so robust and stable that they are often considered cognitive hallmarks of the disorder (Niu et al., 2024).

In contrast, findings on nonverbal executive functions (e.g., visuospatial WM, planning, set-shifting) in DLD are highly variable. Some children show clear EF deficits; others perform within normal ranges (Conti-ramsden et al., 2015). This variability challenges simple causal models. If EF deficits were the primary cause of DLD, then all children with DLD should exhibit impaired EF. Yet, many do not. This suggests a more nuanced relationship in which language and EF may interact and potentially compensate for each other during development. Some children may mitigate language impairments via intact EF resources; others may not, depending on their cognitive profile (Pauls and Archibald, 2016).

Indeed, while both DLD and ASD involve disruptions in language and cognition, the nature and profile of these disruptions differ markedly. DLD is marked by pronounced verbal memory deficits, while ASD is characterized more by social cognition and broad EF challenges. These divergent profiles underscore our central argument: language and cognition are deeply interwoven, and their relationship must be understood within specific developmental contexts. For children with DLD, weakened language skills may hinder the use of language as a cognitive tool, leading to secondary difficulties in memory and learning. However, children with relatively intact EF may develop compensatory strategies. In contrast, ASD children may struggle to engage in language-mediated social learning due to underlying EF and mentalizing impairments—though in some cases, multilingual exposure appears to be beneficial.

### 5.2 ASD: the interplay of language, executive function, and social cognition

ASD is often described as involving a “triple burden” of language impairment, executive dysfunction, and theory of mind deficits. Importantly, these domains do not operate independently but are tightly interrelated in complex ways. Studies have found significant correlations between language abilities (e.g., vocabulary, syntax, pragmatic understanding) and EF performance (e.g., working memory, cognitive flexibility, inhibitory control) in children with ASD (Friedman and Sterling, 2019). The direction of causality is unclear—deficits in one domain may cascade into the others, or shared underlying factors (e.g., atypical brain maturation) may drive co-impairments.

One particularly intriguing line of research has investigated the impact of multilingual environments on children with ASD. Surprisingly, several studies report that bilingual or multilingual children with ASD perform better than their monolingual counterparts on parent-rated measures of EF (e.g., inhibition, shifting) and perspective-taking skills (Ratto et al., 2020; Andreou et al., 2020). They also show fewer core ASD symptoms, such as reduced stereotyped behaviors.

These findings challenge longstanding assumptions. Rather than exacerbating symptoms, multilingual exposure may actually strengthen cognitive control systems and indirectly enhance social functioning. While the current data are largely correlational and based on parent reports, they suggest that language experience can modulate core cognitive features of neurodevelopmental disorders. Importantly, they counter outdated advice to limit language input in ASD children (“Don't confuse them with two languages”), instead pointing toward the potential utility of bilingual environments as part of broader intervention strategies (Hambly and Fombonne, 2012).

### 5.3 Toward integrated interventions

Given the close interdependence of language and cognitive functions in both DLD and ASD, interventions targeting a single domain may be insufficient. Instead, integrative approaches that address both language and EF capacities may be more effective.

For children with DLD, language interventions should go beyond vocabulary and syntax, incorporating supports for attention and memory—such as visual scaffolds or chunking techniques to ease WM load (Montgomery et al., 2024). Similarly, social skills training for children with ASD may be more successful when paired with exercises targeting EF (e.g., turn-taking, rule-switching, response inhibition).

Neuroimaging tools are beginning to play a role in shaping individualized interventions. For example, fNIRS studies in children who stutter have identified atypical blood flow patterns in left frontal language areas, potentially serving as early biomarkers for persistent stuttering (Walsh et al., 2017). Similar approaches could be extended to children with DLD—using fNIRS or EEG to identify weak neural responses to speech sounds or syntactic anomalies. These markers could inform the design of targeted training programs and provide real-time feedback on neural changes.

As the field of educational neuroscience advances, neurobiologically informed interventions are becoming more feasible. Computational modeling, for instance, may help match language games to specific cognitive deficits, optimizing training effects across domains.

In sum, atypical development reminds us that language and cognition are two wings of the same bird. Strengthening one in isolation rarely enables flight. What we need are cross-disciplinary collaborations that unite speech-language therapy, cognitive training, and enriched language environments. Only then can we design interventions that truly harness the brain's capacity for growth—and help every child reach their full potential.

## 6 Cross-cultural and cross-linguistic diversity: expanding the boundaries of language-cognition research

For decades, psychological and cognitive science research has relied disproportionately on WEIRD populations—those who are Western, Educated, Industrialized, Rich, and Democratic (Henrich et al., 2010). This sampling bias raises serious questions about the universality of our theories. Are the observed links between language and cognition truly species-wide mechanisms, or are they artifacts of specific cultural-linguistic environments? To answer this, the field must break free from its reliance on English-speaking, monolingual children and systematically investigate a broader range of linguistic and cultural contexts.

The structural features of different languages (e.g., morphological complexity, writing systems) and the cultural norms of language use can shape cognitive strategies in unique ways. For example, polysynthetic languages such as Inuktitut, which frequently use long compound words, may place different demands on memory and attention compared to analytic languages like Mandarin. Similarly, parenting styles and educational values—such as emphasis on storytelling vs. rule-following—may influence the developmental trajectory and prioritization of specific cognitive skills. We argue that building truly universal theories requires integrating cross-linguistic and cross-cultural diversity into the research agenda.

### 6.1 Commonalities and variability in the language–cognition link

At a macro level, the relationship between language and cognition reflects a fundamental human commonality: across all languages, the acquisition of language provides a framework and set of tools for thought (Ellis, 2019). This principle holds regardless of language type. However, its specific manifestations can vary depending on the structural features of the language and the sociocultural context in which it is used.

For instance, the morphosyntactic features of a language may shape WM demands and processing strategies (Chávez and Auza Benavides, 2017). In highly inflected languages (e.g., Turkish), where words are packed with multiple suffixes encoding nuanced meanings, sentence comprehension may rely more heavily on maintaining and manipulating morphological information—thus training different WM-attention systems. By contrast, in morphologically sparse languages (e.g., Mandarin), children may develop context-sensitive interpretive strategies earlier due to the greater reliance on word order and discourse context.

Cultural values also modulate cognitive development (Wang, 2018). Cultures that prioritize narrative skills may foster children's abilities in memory organization and causal reasoning, whereas cultures that emphasize rule compliance and behavioral restraint may shape inhibitory control (Raver and Blair, 2016). Cross-linguistic studies have found both universality and variability in disorders like DLD. The core impairment in verbal WM is observable across languages (universality) (Niu et al., 2024), but performance on nonverbal EF tasks can differ by language group (variability) (Montgomery, 2002).

Taken together, these findings suggest that while some cognitive effects of language are broadly shared, others are contingent on culturally and linguistically specific experiences. These contextual influences do not undermine the bidirectional relationship between language and cognition; rather, they highlight that this relationship is dynamically shaped by the interplay between universal mechanisms and local adaptations.

### 6.2 Language distance and cognitive control

Not all bilingual experiences are created equal—language distance, defined as the degree of structural similarity or dissimilarity between two languages, can substantially influence the cognitive demands placed on bilingual speakers (Deluca et al., 2019). From a neurocognitive perspective, managing structurally distant languages requires greater engagement of control-related networks—particularly the dorsolateral prefrontal cortex, anterior cingulate cortex, and basal ganglia—due to increased demands on language selection and conflict monitoring (Abutalebi and Green, 2016; Li et al., 2014).

Recent lifespan studies have shown that speakers of more distant language pairs often exhibit enhanced monitoring and attentional switching, especially in aging populations, potentially reflecting sustained recruitment of domain-general executive resources over decades of bilingual experience (Carthery-goulart et al., 2023; Bialystok, 2017). For example, older adult bilinguals managing typologically distant languages demonstrate superior performance in task-switching paradigms compared with those speaking closely related languages, suggesting that long-term exposure to high-conflict language environments may bolster cognitive resilience.

However, language distance interacts with other experiential variables such as code-switching frequency, degree of language mixing, and sociolinguistic context. In immersion settings with high daily switching demands, structural distance may amplify flexibility in task-set shifting, whereas in low-switching contexts, inhibitory control processes may dominate (Wu et al., 2021). Neuroimaging evidence further indicates that distant-language bilinguals show more segregated neural representations in the language network, while close-language bilinguals exhibit greater overlap, potentially influencing control strategies (Pliatsikas, 2020).

Another emerging direction is the study of multilinguals, where the acquisition of a third or fourth language creates a natural gradient of language distances. Research indicates that the proximity of a new language to one's existing languages influences not only vocabulary acquisition but also adaptive changes in working memory capacity and control mechanisms (Engel de abreu and Gathercole, 2012). This perspective extends beyond traditional binary bilingual comparisons, emphasizing the complex, multidimensional nature of language experience in shaping cognitive control.

By situating language distance within a broader neurocognitive and experiential framework, future research can move beyond simple dichotomies and toward integrative models that capture how structural, functional, and sociocultural factors jointly modulate the bilingual cognitive profile.

### 6.3 Methodological challenges and opportunities in cross-cultural research

Comparing cognition across cultures is like measuring with different rulers—unless carefully calibrated, results may be misleading. Traditional standardized assessments often embed cultural biases. For instance, vocabulary tests are highly sensitive to formal education and cultural exposure, putting children from rural or marginalized backgrounds at a disadvantage.

To mitigate this, some researchers advocate for processing-dependent measures that minimize the influence of acquired knowledge (Quinn et al., 2011). Tasks such as digit span and non-word repetition are less tied to cultural content and may allow for fairer cross-group comparisons.

However, even these measures are not entirely culture-free. For example, the number of commonly used digits in a language and the phonological familiarity of nonwords can subtly influence memory performance (Grivol and Hage, 2011). This is known as the “lexicalization effect”—pseudo-words that resemble real words or high-frequency phoneme sequences are easier to recall (Martínez-tomás et al., 2025). Thus, in cross-cultural assessments, researchers must both minimize cultural loading and statistically account for any residual biases.

A promising future direction lies in developing truly cross-linguistic cognitive assessment tools. For instance, using abstract pattern-based tasks (that don't require language) or creating language-specific test versions calibrated for difficulty equivalence. Large-scale international collaborations can help normalize these tools across diverse samples, setting global benchmarks.

In summary, addressing cultural bias is not about abandoning comparisons but about making them smarter. By acknowledging the cognitive diversity of humanity while seeking shared underlying dimensions, we can build theories that are both broadly generalizable and sensitive to local realities.

## 7 General discussion and future directions

Language is not merely a passive tool stored within the cognitive system—it is an active, dynamic force. It serves as an “architect” of cognition, shaping the very mechanisms it helps enact throughout development. In this review, we conceptualize language as a bi directionally interactive and culturally embedded cognitive system, wherein structural and experiential factors jointly remodel the brain's architecture over the lifespan (Bialystok, 2017; Li et al., 2014). This perspective calls for a shift away from modular, unidirectional models of causality toward more integrative, dynamic, and context-sensitive frameworks in understanding the neurobiological foundations of language.

Importantly, culture is positioned here as a contextual moderator—not as an alternative causal direction—but as a factor that shapes the magnitude and manifestation of language–cognition links. For instance, differences in communicative norms, educational practices, and societal bilingualism policies can modulate the cognitive benefits associated with language experience (Nuri, 2024).

Building on the integrative framework presented here, we propose several promising directions for future investigation:

1) Developmental Cascade and Causal Testing Longitudinal cross-lagged or experimental studies are needed to clarify the causal pathways between language and EF. Pioneering studies have already demonstrated partial causal links in early childhood, providing a methodological blueprint (Engel de abreu and Gathercole, 2012). For example, an intervention could contrast a language-enrichment group (enhanced input in vocabulary and syntax) with an EF-training group (memory, inhibition, shifting games), tracking untrained domains over time to test co-development hypotheses. Prior studies indicate that language-focused interventions in preschoolers can yield transfer effects to EF measures, particularly in working memory and cognitive flexibility (Engel de abreu and Gathercole, 2012). These findings support—but do not yet fully specify—the developmental timing and mechanisms of transfer, leading to larger-scale, stage-specific trials.
2) Neurointerventions and Network Plasticity Noninvasive neuromodulation (e.g., tDCS, TMS) can probe causal links in the brain. For instance, stimulating or inhibiting subregions of the left inferior frontal gyrus (IFG) may reveal dual effects on language and domain-general control tasks. Evidence from noninvasive stimulation studies shows that targeting the dorsolateral prefrontal cortex (DLPFC) modulates syntactic processing efficiency and sentence comprehension accuracy, supporting its causal role in integrating language and executive functions (Hertrich et al., 2021; Hussey et al., 2015). Combining these protocols with functional neuroimaging and machine learning could track how intensive bilingual or syntactic training reorganizes brain networks, detecting shifts in the topology of the MD and language networks.
3) Language as a Pillar of High-Level Cognition To validate the “syntax supports ToM” hypothesis, neurodevelopmental studies should integrate EEG and eye-tracking to examine neural responses when children process embedded mental-state sentences. Foundational work has illuminated how syntactic complexity interfaces with neural systems for higher-order cognition, including ToM and discourse processing (Bourguignon, 2023). Further, studies in sign language users and minimally verbal children with ASD can test whether alternative modalities (e.g., signed complement clauses, visual-symbol systems) facilitate ToM acquisition, informing whether syntax is necessary or merely sufficient for representing others' minds.
4) Integrative Cross-Linguistic Models Large-scale international collaborations should examine cognition under comparable paradigms across typologically and culturally diverse languages (e.g., English, Mandarin, Turkish, Spanish). By incorporating multilevel modeling, we can partition variance due to linguistic structure, cultural practices, and socioeconomic status, clarifying universal versus culture-specific patterns. This approach also directly operationalizes our theoretical stance on culture as a moderator, enabling empirical tests of how sociocultural context shapes the strength of bidirectional language–cognition links.
5) Real-World Applications in Education and Clinical Practice Theory must inform practice. “Bilingual–EF fusion curricula” could integrate language learning with EF-enhancing tasks in schools, while clinical programs for DLD could combine speech—language therapy with EF game-based interventions. Neuroimaging could track both behavioral and neural changes, testing whether integrated programs outperform traditional methods.

In sum, we are at the cusp of a more profound understanding of the language—mind relationship. We acknowledge the rich theoretical history and neuroscientific work in this domain and view our synthesis as a bridge between these traditions and emerging data-driven models. Through interdisciplinary collaboration, cross-cultural engagement, and rigorous empirical testing, we can move closer to a comprehensive picture of how this “architect” builds the magnificent edifice of the human mind.
